# Nonthermal Biocompatible Plasma Inactivation of Coronavirus SARS-CoV-2: Prospects for Future Antiviral Applications

**DOI:** 10.3390/v14122685

**Published:** 2022-11-30

**Authors:** Ihn Han, Sohail Mumtaz, Eun Ha Choi

**Affiliations:** 1 Department of Plasma Bio-Display, Kwangwoon University, Seoul 01897, Republic of Korea; 2 Plasma Bioscience Research Center (PBRC), Applied Plasma Medicine Center, Kwangwoon University, Seoul 01897, Republic of Korea; 3 Department of Electrical and Biological Physics, Kwangwoon University, Seoul 01897, Republic of Korea

**Keywords:** nonthermal plasma, COVID-19, SARS-CoV-2, NBP viral inactivation, coronavirus disinfection

## Abstract

The coronavirus disease (COVID-19) pandemic has placed a massive impact on global civilization. Finding effective treatments and drugs for these viral diseases was crucial. This paper outlined and highlighted key elements of recent advances in nonthermal biocompatible plasma (NBP) technology for antiviral applications. We searched for papers on NBP virus inactivation in PubMed ePubs, Scopus, and Web of Science databases. The data and relevant information were gathered in order to establish a mechanism for NBP-based viral inactivation. NBP has been developed as a new, effective, and safe strategy for viral inactivation. NBP may be used to inactivate viruses in an ecologically friendly way as well as activate animal and plant viruses in a number of matrices. The reactive species have been shown to be the cause of viral inactivation. NBP-based disinfection techniques provide an interesting solution to many of the problems since they are simply deployable and do not require the resource-constrained consumables and reagents required for traditional decontamination treatments. Scientists are developing NBP technology solutions to assist the medical community in dealing with the present COVID-19 outbreak. NBP is predicted to be the most promising strategy for battling COVID-19 and other viruses in the future.

## 1. Introduction

Viruses are the world’s most abundant and varied microorganisms. They’ve been on the planet for billions of years [1,2,3], have evolved to a variety of environments, and can now be found in all ecosystems. Pathogenic viruses cause tens to hundreds of millions of plant, animal, and human infections each year, resulting in significant agricultural losses and countless fatalities. To improve one’s quality of life, it is necessary to inactivate dangerous viruses. The human coronavirus disease-2019 (COVID-19) is an epidemic infection created by SARS-CoV-2 (severe acute respiratory syndrome coronavirus-2), and it was initially spotted in Wuhan, Hubei province, China. This infection has extended throughout the world and is also progressing during the present pandemic. 

COVID-19 disease is a new infectious disease caused by SARS-CoV-2. As an airborne virus, it can infect humans through respiratory droplets in the same way as common colds and the flu [4,5,6]. The virus’s rapid spread has quickly reached a pandemic status, threatening almost the whole world population and affecting millions with the clinical manifestations and morbidities associated with the disease it causes, COVID-19. SARS-CoV-2 caused a once-in-a-century pandemic, and studies have shown that the infectious virions can survive on various surfaces for several hours (e.g., plastic, metals, and cardboard) [4]. Surface contamination poses a significant risk of SARS-CoV-2 transmission between people, and breaking the transmission cycle by developing new inactivation methods is critical. Modern drug discovery practices include a wide range of theoretical and computational approaches, collectively known as computer-aided drug design (CADD). CADD techniques have played a significant role in the development of several drugs that are currently being tested in clinical situations. These techniques have been developed in combination with experimental strategies used in drug development [7]. The recent outbreak of COVID-19, a novel coronavirus disease, calls for and welcomes potential treatment plans utilizing the available pharmaceuticals. Computer-aided drug design methods are very effective for quickly identifying potential drug repurposing candidates, particularly after the precise 3D structures of important viral proteins are resolved [8,9,10].

In the realm of viral inactivation, nonthermal biocompatible plasma (NBP) has emerged as an innovative, effective, and safe approach over traditional ones. NBP can be used to inactivate viruses in an environmentally acceptable manner and can activate a variety of animal and plant viruses in a variety of matrices. The reactive species have been demonstrated to be responsible for viral inactivation. Disinfection methods based on NBP technologies provide an exciting solution to many of these difficulties because they are simply deployable and do not necessitate the resource-constrained consumables or reagents required for traditional decontamination practices. NBP technologies have shown considerable potential in a variety of medical applications ranging from wound healing and cancer treatment to sterilization approaches to reduce virus transmission by airborne and fomite transfer. We hope that this review will give readers a platform to examine the progress made in the fight against COVID-19 through NBP technology.

## 2. Virus Deactivation by Radiations

**Figure 1:**
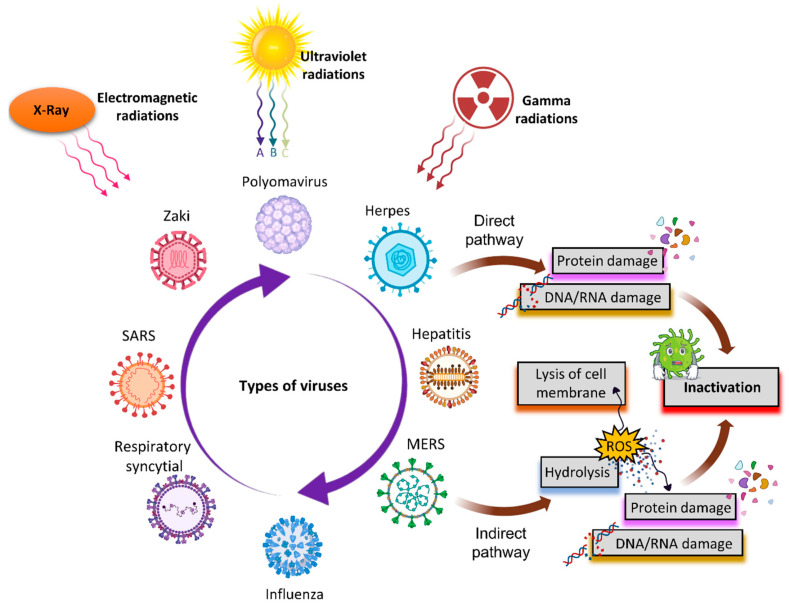
The illustration of the direct and indirect impacts of several types of radiation on microorganisms.

Radiations are well known to have several effects when interacting with biological systems [11,12,13,14,15,16]. The mechanism by which an electromagnetic field affects biological systems was presented well [11]. For the decontamination of various virus species, a variety of physical approaches have been used, including electromagnetic irradiation, X-rays, UV, and gamma radiations [17,18,19,20,21]. Radiation production is an ongoing subject of study [22,23,24,25,26,27,28,29,30,31,32] that is beneficial to our modern lives. Figure 1 graphically depicts the direct impact and mechanism of employing radiations to disinfect microorganisms. Irradiation is a low-energy, ecologically friendly, and safe way of killing viruses under carefully regulated conditions with little molecular modifications, which is particularly significant in the manufacture of biological reagents. The sun’s UV radiation in the atmosphere is the most effective natural germicide. Far-UVC light, with a wavelength range of 207–222 nm, on the one hand, probably eliminates germs without harming their natural equivalents [33]. 

UV absorption and viral inactivation are both aided by nucleic acids found within pathogens. UV light inactivates and tabulates the susceptibilities of a wide range of viruses, including those with double/single-stranded RNA or double-stranded DNA genomes, at a wavelength of 254 nm [33,34]. SARS-CoV-2 can be successfully inactivated by UVC irradiation, according to a recent study [35,36,37]. Recent research has also emphasized the use of UV radiation to decontaminate N95 respirators to assure COVID-19 safety [38]. 

Gamma radiation is ionizing radiation with the smallest wavelength and most of the energy in the electromagnetic spectrum that is capable of inactivating DNA and RNA viruses [39,40,41]. The breakdown of DNA/RNA by radiolysis or genetic material cross-linking is assumed to be the main process behind the inactivation of viruses by irradiation [42]. In other words, it can either directly break down the DNA helix or produce free radicals that cause DNA damage [43]. The use of gamma irradiation to sterilize harmful organisms in the environment is efficient. Gamma radiation is commonly used in the sterilization of medical devices, injectable goods, and food samples due to its great decontamination capabilities [44]. Because of the present global COVID-19 epidemic, numerous researchers have looked at how effective gamma radiation is at inactivating the virus. Gamma radiation has been claimed to play a key role in vaccine manufacturing via viral inactivation since it has previously demonstrated efficacy in inactivating other enveloped viruses [42,45,46]. It was recently discovered that the sample volume and protein content of the sample influenced viral inactivation by gamma irradiation using a surrogate virus [40]. X-rays can stop the virus from regressing by stopping cellular division and causing pathological alterations that eventually kill the virus [47]. Ionizing radiation has proven to be a highly successful approach to disinfecting gloves, surgical masks, and other items in the case of SARS-CoV-2 [48,49,50,51,52].

## 3. Virus Deactivation Using Emerging Nonthermal Biocompatible Plasma (NBP) Technology

### 3.1. Introduction of Plasma

**Figure 2:**
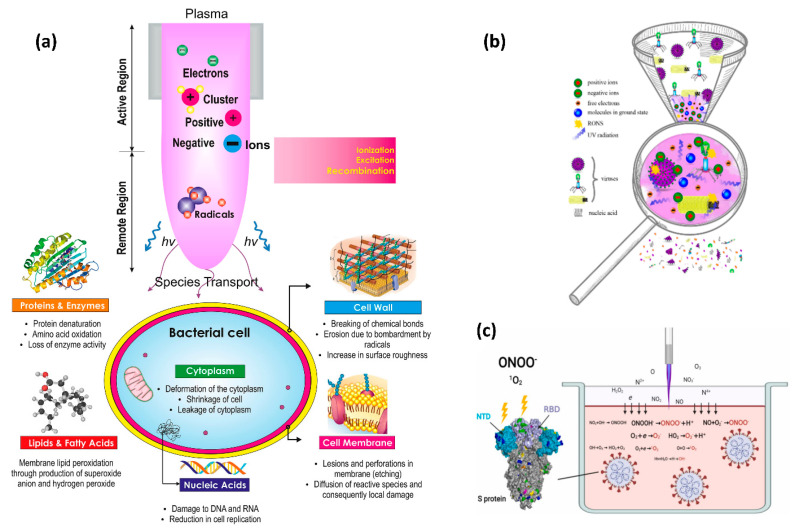
(**a**) A diagram depicting the action of cold plasma on microbial cell structures, resulting in functional loss and sterilization [54]. (**b**) The mechanism of viral inactivation by plasma [55]. (**c**) The mechanism through which RONS produced in Ar-based NBP damage S protein. Plasma jet-generated reactive species dissolve into liquid and cross-react in the liquid phase. Predominant RONS ONOO^−^ and O_2_^−^ oxidized tyrosine, tryptophan, and histidine at RBD and NTD, thereby impair RBD’s capacity to bind to cell receptor ACE2 and NTD’s function. After 3 minutes of cold atmospheric plasma (CAP) or NBP treatment, the viral genome remains intact. Reprinted with permission from [56].

Plasma is the most common kind of matter, accounting for 99% of the entire universe. The plasma state comprises the sun and other stars, galaxies, solar winds, lightning, and the aurora borealis. The most well-known man-made uses of plasma include plasma televisions, as well as neon and fluorescent lighting. As seen in Figure 2, plasma is composed of free electrons, atoms, and molecules in neutral, ionizing, and/or excited states, as well as reactive species such as reactive oxygen species (ROS) and reactive nitrogen species (RNS). The plasma, made up of a variety of gases, is a large source of UV and vacuum UV radiation [53]. Plasma is a unique material-treatment technology since it can utilize a single ingredient or a mixture of elements.

### 3.2. Thermal and Nonthermal Plasma

Two basic groups of plasma systems are typically characterized: thermal plasmas and non-thermal plasmas, in which the temperatures of the various plasma species differ. Due to growing interest in industries like aerospace, microelectronics, automotive, material processing, metal melting and welding, plasma chemical synthesis, vapor deposition, plasma, arc spraying, and waste destruction during the past few decades, thermal plasma technology has advanced [57]. The major phenomena involved in typical atmospheric plasma devices are Joule heating and thermal ionization [57]. These devices are made possible by arcs or radio frequency (RF) inductively coupled plasma discharges. The main limitations of thermal plasmas include low excitation selectivity and extremely high gas temperatures, as well as stringent quenching requirements and electrode issues that restrict the energy efficiency and practicality of thermal plasma sources. Thermal plasmas are nearly fully ionized plasma in which all particles have about the same temperature. Thermal plasma was also known as hot plasma or equilibrium plasma. Based on the demands, the equilibrium plasma was used in a variety of applications [58,59]. On the other hand, nonthermal plasma contains light electrons, which have much higher temperatures than heavy atoms and molecules and are often close to room temperature. Due to their high selectivity in plasma chemical processes and their ability to function well at low temperatures without quenching, non-thermal plasmas, such as low-pressure glow and RF, microwave discharges, dielectric barrier discharges, and laser-produced plasmas, have been used. Non-thermal atmospheric pressure plasmas have more recently been researched for a range of industrial and medical applications and are known as NBP or plasma medicines [58,60,61,62,63,64,65,66]. The NBP was also known as a cold plasma or non-equilibrium plasma. NBP is appropriate for treating a wide range of biological materials, including solids, liquids, and aerosols because it is at room temperature at the site of application. Low pressure and atmospheric pressure are two different types of NBPs that offer a wide range of applications [58,59,62,64,67,68,69,70,71]. Low-pressure plasma spreads across a vast volume, but high-pressure plasma is restricted to the volume where substantial electric fields exist [59]. Electric discharges are commonly used for the sustainability of plasma. The temperature of the gas is normally unchanged, but because of the presence of reactive species, the chemical reactivity is much higher than the source gas. Because of practical reasons, atmospheric pressure plasma has been employed in biological systems [55,72]. Numerous in vitro and in vivo investigations have demonstrated the positive effects of plasma. In several clinical studies [73,74], NBP was also shown to be safe for people and is well-known as a plasma medicine [58,75,76].

### 3.3. Generation and Role of Reactive Species in Anti-Viral Responses

ROS are important cell-signaling molecules for normal biological development, and they are often generated by both external and endogenous stimuli. However, the production of ROS can harm a variety of cellular organelles and activities, disrupting the cells’ normal functioning [54,77,78,79,80]. When measuring a cell’s oxidative stress levels, it is important to remember if an enhanced oxidant status causes biomolecule damage and defines the critical threshold for cellular functions via redox signaling [77,81]. ROS may be produced by a variety of medications as well as traditional physical methods like X-rays and gamma radiation. A broad range of reactive species is generated in cold plasma [82,83,84], depending on several factors such as the feeding gas, the target material’s configuration, the energy source, and the distance between the target and plasma source during plasma discharges. Free energetic electrons produced by plasma have energies high enough to electrically excite, dissociate, and ionize molecules, resulting in more than 80 distinct species in humid air [85,86]. Plasma-induced ROS play a vital role in the deactivation of various pathogens. That is why NBP has become a future hope for the deactivation of viruses [87].

Because of the interaction between free electrons and the feeding gas molecules, primary reactive species, such as nitric oxide radicals (NO), excited nitrogen (N), hydroxyl radicals (OH), superoxide radicals (O_2_*), and singlet oxygen (^1^O_2_), are directly generated in the plasma discharge zone. Although these species have a short lifespan, their concentration in the discharge area is very significant. More importantly, multiple studies [88,89,90] reveal that the amount of OH radicals generated in the ambient air is quite high. In the ambient environment, the species are further transformed into long-lived reactive species such as hydrogen peroxide (H_2_O_2_), nitrite (NO_3_), ozone, and nitrate (NO_2_). The principal reactive species may react with molecules in the surrounding air, resulting in stable species. The H_2_O_2_ is the most dispersible and stable of the three within the water, whereas NO_3_ and NO_2_ are converted to NO_3_^−^ and NO_2_^−^, respectively. It’s worth noting that the chemistry underpinning the formation of reactive oxygen and nitrogen species [43] varies significantly depending on the plasma discharge circumstances [91].

The plasma-activated medium (PAM) can be made by exposing a biological liquid to electrical discharge under ambient circumstances [92,93,94]. When the plasma is formed in the natural environment, it produces a substantial array of reactive species. These RONS are key species that allow NBP technology to be used in a wide range of biological applications. This approach might be useful for healing serious wounds or decontaminating infectious areas. The plasma is immediately exposed to biological solutions in the off-site approach, which are used for specialized biomedical applications. Variations in the supplying working gas, flow rate, and applied voltage, for example, can affect the levels of reactive species formed. Short-lived reactive species can be used in situ, whereas long-lived reactive species can be stored in PAM and used later.

The viral inactivation mechanisms might be ROS, RNS, or a composite of both, depending on the treatment method, circumstances, and plasma supply. Because transport constraints are extremely significant for plasma-based decontamination, the species that are ultimately responsible for the viral inactivation may rely not only on the dominant species generated by the plasmas but also on the environmental and treatment circumstances. Consequently, this may introduce certain system- and treatment-specific characteristics to the viral inactivation processes by NBP.

## 4. Disinfection of Viruses Inside Water by Using NBP

Even though water is necessary for life, coronavirus has been shown to be able to survive there for days, and it serves as a typical means of viral dissemination [95,96,97,98,99]. The WHO claims that the main way COVID-19 transmits from person to person is by airborne droplets that are emitted when an infected person coughs, sneezes, or even talks. If someone inhales these droplets from an infected individual, they may get COVID-19 [100]. Through recombination, reassortment, and mutation, viruses may adapt to new environments, from air to aquatic life, and survive in water [101]. Due to the COVID-19 epidemic, interest in UV disinfection and sterilization technologies has increased. In principle, prolonged or repeated exposure to UV radiation is never actually safe for people. UV rays, both direct and indirect, can damage the skin and eyes of people. UV radiation is a potent energy source, and due to its characteristics, it is lethal to germs and viruses while being toxic to people. As shown in Table 1, several conventional and alternative technologies were used to sterilize water or destroy the viruses present in it. The current technologies and procedures have the ability to render viruses inactive, but they also come with substantial disadvantages and limitations for a wide range of applications.

In contrast to other disinfection methods indicated in Table 1, NBP sometimes referred to as cold plasma, joins the decontamination procedures as a novel, effective, cost-free, safe, and environmentally friendly substitute for inactivating viruses [55,102]. Like many other strategies, the NBP strategy needs improvement. Understanding the factors that affect NBP’s antiviral activity inside water is crucial [103]. According to several studies, a number of factors are significantly presented which are effective in NBP [104]. The NBP potential is also greatly influenced by the pH and conductivity of the water since these factors are closely connected to ion generation in the aqueous medium and, consequently, to how well the reactor produces active species in water. Conductivity, pH, and viral inactivation effectiveness are all inversely correlated. In response to the administration of NBP, nitrite species develop in the water, lowering the medium’s pH and increasing conductivity as a result of the interactions between the ionic species they produce [105]. When it comes to the inactivation of viruses inside water, salinity, and temperature, also have a substantial role [106]. Salinity and temperature are associated because salinity has a greater impact at lower temperatures. According to research, temperature has a more significant influence than salinity alone in reducing viral persistence in water [107]. Viral persistence at low temperatures can be increased by high salt. However, minute temperature changes have nonlinear effects on the persistence of viruses and less NBP inactivation efficiency. Furthermore, the working gas in NBP technology and device types are also important to achieve better inactivation inside water. The RONS generated by NBP successfully inactivated the SARS-CoV-2 inside the water. This is the process by which the S protein is harmed by RONS generated in Ar-based NBP. Reactive species produced by plasma jets dissolve into liquid and interact with one another inside. Tyrosine, tryptophan, and histidine were oxidized at RBD and NTD by the predominate RONS ONOO^−^ and O_2_, which affected RBD’s ability to bind to the ACE2 cell receptor and NTD’s functionality. The viral genome remained unaltered after 3 minutes of treatment with NBP (Figure 2c) [56].

The prevalence of numerous COVID-19 variants has raised interest in the use of NBP technology for viral inactivation in water. NBP technology is one approach that may be used to overcome problems with water purification. There are questionable arguments with NBP, which need more research and development for an answer. Therefore, further research and debate on this particular technology’s limitations are needed in order to make its use financially viable. Additionally, techniques for inactivation based on ROS and RNS may provide promise in the future.

## 5. Applications and Mechanism of NBP for Virus Deactivation

Plasmas are employed in a variety of industries, mostly to customize the surfaces of solids (e.g., oxidation, cleaning, nanostructuring, and binding distinct atom/molecule groups), but they are also used to destroy microbes such as viruses. Plasma can be used to treat liquids as well; however, inactivating viruses in a liquid medium is more difficult compared to a solid media because plasma cannot be preserved in liquids it can only be found in gaseous bubbles within the liquids or just above the surface of the liquid. RONS interacts with the bubble or liquid surface, where many disintegrate, depending on where they are generated. They can then disperse through the liquid, perhaps interacting with the virus. UV light also penetrates liquids with a penetration depth that varies substantially depending on the wavelength, concentration, and type of contaminants [108]. There are several ways for detecting long- and short-lived RONS in liquids [109], but they are not commonly utilized by researchers studying viral annihilation. Many publications provide discharge parameters (voltage, current, and power) rather than plasma parameters (reactive species concentration), which are required to compare different plasma sources. As a result, the plasma–virus scientific domain is still in its early stages. Research on dielectric barrier discharge (DBD) devices and PAS on bacteriophages was undertaken in 2018 to investigate the mechanism of the influence of plasma application in viruses [110]. The reactive species produced by plasma treatment were shown to damage DNA and proteins, disrupting viral cells. 

**Figure 3:**
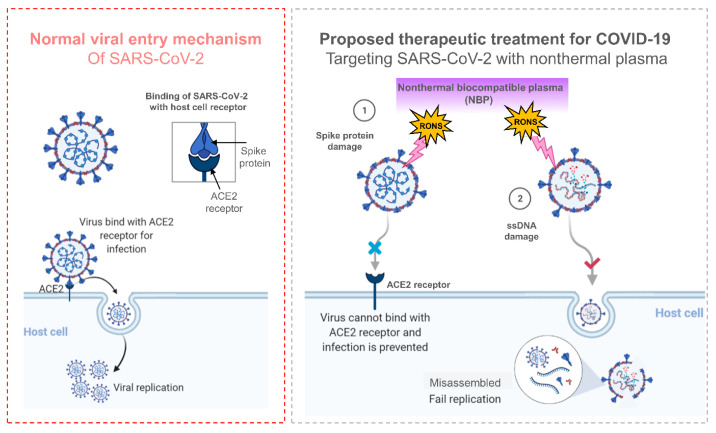
The mechanism of normal viral entry of SARS-CoV-2 to a host cell and the concept of its inactivation by utilizing NBP. Various RONs produced by NBP bind directly to the surface of virus particles to capture hydrogen atoms/ions and cause damage to the membrane proteins of the virus. It refuses to let the virus enter the lung cells caused by spike proteins bound to membranes, which do not bind properly to ACE-2 receptors. On top of that, the capsid proteins that protect RNA are decomposed and the molecular binding of RNA is broken, and even if the virus penetrates through the alveolar plasma membrane, contact viral components are not synthesized in lung cells due to genetic information damage. NBP is possible with new treatments against SARS-CoV-2 infection by these two-way solutions.

A decade ago, Yasuda et al. [111] proposed a biological technique to quantify the in vivo DNA damage caused by bacteriophage lambda viruses exposed to air plasma. Xia et al. conceived and built a packed-bed DBD plasma reactor that efficiently deactivated MS2 bacteriophages in aerosols utilizing a nominal pressure drop across the reactor for air sterilization against airborne bacteriophages. This method is beneficial for preventing viral infections from spreading through the air [112]. Several other researchers have discovered that plasma causes considerable protein oxidation of the viral coat or capsid. Through the action of ROS, plasma treatment makes bacteriophages dormant. However, further research is needed to figure out whether plasma operating parameters promote protein inactivation or viral DNA damage. A variety of NBP sources can fully inactivate or greatly limit the infectivity of multiple humans, animals, and plant harmful viruses, and a mechanism was proposed recently [55], as shown in Figure 2b. Furthermore, the process by which RONS is formed in NBP is based on Ar damage to the S protein. Reactive species created by plasma jets dissolve in liquid and cross-react in the liquid phase. RONS ONOO^−^ and O_2_^−^ oxidized tyrosine, tryptophan, and histidine at RBD and NTD, limit RBD’s ability to bind to the cell receptor ACE2 and NTD’s function as indicated in Figure 2c. Figure 3 illustrates the mechanism of inactivation of SARS-CoV-2 by using NBP. The spike proteins of the SARS-CoV-2 attach to the ACE2 receptor during normal viral entry and so enter the host cell for viral replications. When SARS-CoV-2 was exposed to NBP, the spike proteins were damaged, preventing the virus from binding to the ACE2 receptor and preventing infection. On the other hand, NBP also damages the DNA/RNA, which restricts the SARS-CoV-2 for viral replication.

Vaccine production and supply are critical components of any vaccination approach since they ensure that a vaccine is extremely efficient in avoiding viral infection or virus-associated illnesses. The role of NBP in each of these critical components of vaccine development is now being investigated owing to the properties of NBP sterilization in air, liquid, and surfaces. The NBP therapy is expected to have a wide margin of safety, as evidenced by animal and human research [113,114], which found no notable acute or long-term adverse effects on therapeutic NBP application to the skin. Because of the focused nature of NBP application, it will be impossible to give NBP to all the cells that host the virus in infections caused by viruses that disseminate to various tissues, organs, and compartments (e.g., HIV-1 and SARS-CoV-2).

## 6. Conclusions and Perspectives

Although it is difficult to predict how the ongoing COVID-19 epidemic will unfold, effective countermeasures must be developed and implemented as soon as possible. NBP-based disinfection treatments operate by interfering with the survival of the virus’s critical structural and/or functional components and life cycle, using either direct application or an NBP-activated medium. To increase vaccine distribution and/or immunity conferral, plasma might be utilized in combination with current preventative methods such as immunization. These treatments offer significant advantages over typical sterilizing procedures for SARS-CoV2. When an infectious disease epidemic occurs, NBP-based solutions may be immediately deployed and used without the need for costly consumables, continuous supply chains, or expensive and dangerous chemicals. While NBP discharges efficiently decontaminate SARS-CoV-2 bioaerosols, direct NBP application or NBP-activated media can safely and effectively disinfect a variety of surfaces, including skin and medical PPE. NBP addresses several shortcomings of conventional antiviral and sterilization methodologies and has the potential to be an effective antiviral technology. When applied uniformly and directly to exposed surfaces, UV-C radiation, for instance, has a limited depth of penetration and lacks diffusion and turning functions [115]. 

The fluidity of plasma could be used in solutions to solve this issue. A high-energy electron beam has been used to inactivate coronavirus on cold chain food outer packaging, according to studies, and has controlled penetrating depths [116,117,118]. Therefore, electron beams could be used to simultaneously inactivate viruses inside and outside of objects if combined with plasma [119,120]. Additionally, SARS-CoV-2 is primarily spread through the air. It can be difficult to set up NBP sterilization devices for large spaces with high airflow to meet the requirements for sufficient exposure time to effectively eradicate the virus. Additionally, NBP-enabled biomedical technologies present special chances to improve antiviral therapies. According to studies, NBP increases the body’s immune response, which can then be used to treat viral pathogens within the body rather than just applying NBP directly [121]. Plasma may be used to increase the antigenicity of viruses in vaccine preparations after inactivation or can be applied topically to promote the activity of antigen-presenting cells, enhancing immune responses to immunotherapies or during vaccination. Additionally, different manufacturing processes can produce various NBP types, enabling the customization of NBP technologies for particular biomedical scenarios. NBP-based strategies for producing inhaled NO and for treating critically ill and intubated patients may also be taken into consideration, even though the care of COVID-19 patients continues to be primarily supportive. Finding the right reactive species and efficient delivery strategies that enable strong preventative measures for enhancing infection control and the clinical translation of new treatments for COVID-19 and in consideration of future pandemics will be a significant challenge for the NBP and medical research communities going forward [121,122,123]. Furthermore, there are several promising biomedical applications for NBP. 

One major benefit of NBP is that they can be readily produced from air, water, and electricity, providing a sterilization solution at a lower cost without the expense and logistics of maintaining expensive and robust supply chains, which are required for conventional methods that rely on consumables like alcohol and hydrogen peroxide [105,113,124]. In the end, its use should reduce the number of infections in humans, animals, and plants, as well as lower economic and biological costs. NBP can inactivate airborne viruses since it is produced with less energy and has an active electron at a significantly higher temperature than bulk gas molecules [125]. Setting the right parameters and selecting treatment periods that allow particles to interact with the contaminated material is crucial when employing NBP for virus inactivation. It has been established that ROS and RNS have an impact on capsid proteins and/or nucleic acids, which results in virus inactivation. The development of more precise techniques will reveal which plasma particles are essential in each experiment and exactly how they impact viruses. Water decontamination, we feel, is one of the domains of viral inactivation where the plasma might be a more important advance. For human use and/or agricultural reasons, NBP might inactivate troublesome enteric viruses and hardy plant viruses. In any event, the possible detrimental genotoxic and cytotoxic consequences of plasma-activated water on humans and plants must be assessed first. However, the NBP technology for viruses is in its early stages and needs further exploration. To clarify which plasma particles are most significant, as well as how they affect viruses, more accurate approaches are desperately required.

## Figures and Tables

**Table 1:** Summary of methods to inactivate viruses inside water.

Methods	Benefits	Limitations
Chemicals	-Easy to use and energy-efficient -Quick responses -Consistent performance	-Insufficient to regulate and remove viruses from water -Harmful and risky for human health -Quick volatilization, which necessitates particular storage tanks
Ultraviolet (UV)	-UVC technology can get rid of bacteria and viruses on surfaces, airborne infections, and particles. -Eliminates of bacteria, viruses, mold spores, and germs.	-Due to its propensity to cause eye damage, UVC exposure is dangerous to people
Ozone	-Highly effective at killing microorganisms	-Could be expensive if ozone disinfectant is used -Limited research on ozone and SARS-CoV-2
Heat inactivation	-Effective with high rates of inactivation -Possibility of the temperature selection for the inactivation of different viruses	-When additional heat is needed, running costs are significant and energy-intensive -Inadequate technology -Threats to public health
Membranes	-Partially effective in preventing infections -Automatization is conceivable	-High chance of clogging -Expensive consumables
Photocatalytic	-Effective and safe for viral disinfection	-High expenditures for disinfecting big effluent
Adsorption	-Effective for viral inactivation in a short amount of time -Simple to use	-The pH adjustments are necessary -Filtration device is necessary -Frequent vessel cleaning is required
Nonthermal Biocompatible plasma (NBP)	-Broad disinfection spectrum -No toxic threat -Effective method for inactivating germs and viruses -Cocktail of many reactive species (RONS) -Ability to inactivate any virus or its variants	-Not accessible on a large scale commercially -The processes that cause viral inactivation in water are not well understood -The particular reactive species which might have a significant role to inactivate viruses is not clearly identified
